# Higher abundance of the vector Aedes aegypti in rural areas than in urban areas in Managua, Nicaragua

**DOI:** 10.21203/rs.3.rs-6059011/v1

**Published:** 2025-03-12

**Authors:** Harold Suazo Laguna, Jacqueline Mojica Díaz, María M. Lopez, Angel Balmaseda, Eva Harris, Josefina Coloma, Jose G. Juarez

**Affiliations:** Sustainable Sciences Institute; Sustainable Sciences Institute; Sustainable Sciences Institute; Sustainable Sciences Institute; University of California; University of California; Sustainable Sciences Institute

**Keywords:** Aedes aegypti, mosquitoes, urban, rural, Nicaragua

## Abstract

**Background:**

*Ae. aegypti* is the primary vector of dengue, chikungunya, and Zika viruses, traditionally associated with urban environments. However, its presence and abundance in rural settings remain understudied. This study compares *Ae. aegypti*populations between rural and urban communities in Managua, Nicaragua, across different seasons over multiple years.

**Methods:**

Entomological surveys were conducted in 500 randomly selected households (250 rural, 250 urban) during the rainy and dry seasons of 2022 and 2023. Immature mosquitoes were collected from water-holding containers, and adult mosquitoes were sampled using aspirators. Entomological indices, including *Stegomyia*, pupal, and adult indices, were compared across seasons and localities.

**Results:**

All entomological indices were significantly higher in rural communities than in urban areas across both years and seasons. Rural households had greater mosquito densities, with pupal productivity concentrated in large water storage containers. Adult mosquito collections confirmed a greater *Ae. aegypti* presence in rural areas, suggesting sustained transmission risk. We observed pupal thresholds in water-holding containers for female adult collections.

**Discussion:**

Contrary to the conventional view of *Ae. aegypti* as an urban mosquito, our findings highlight its substantial presence in rural settings, likely driven by water storage practices and environmental conditions. These results align with findings from other regions reporting high mosquito abundance in rural areas, challenging assumptions about urban dominance.

**Conclusion:**

Rural areas play a crucial role in sustaining *Ae. aegypti* populations. Vector control strategies should target both rural and urban communities, with seasonally tailored interventions to mitigate disease transmission risks.

## Background

Arthropod-borne viruses (arboviruses) such as dengue, chikungunya and Zika viruses are the most important human viral diseases transmitted by mosquitoes (1). The public health impact of these diseases has increased over the past decades, especially due to dengue virus (DENV), which has spread to new geographic locations and places more than 4 billion people at risk of infection globally (2). These arboviruses are transmitted by mosquitoes of the genus *Aedes*, with *Ae. aegypti* (L.) being the main vector for disease transmission (3). *Ae. aegypti* is a highly anthropophilic mosquito, with its global distribution expanding due to the effects of climate change (4). As such, it is critical to fully understand how local populations of *Ae. aegypti* are modulated by spatiotemporal factors such as location and seasonality over time (5–7). However, several regions of Central America remain with scarce information regarding seasonality data for *Ae. aegypti*.

In Nicaragua, dengue has been the most significant mosquito-borne disease since 1985, when the first epidemic was recorded, resulting in over 17,000 cases and seven deaths (8). Over the past decades, DENV has caused epidemics every 2–3 years (9–11). Previous work in Managua has shown the potential of improving ongoing vector control of *Ae. aegypti* by means of community engagement and source reduction (12–14). These studies have also provided information regarding productive containers for immature stages, seasonal trends, and risk factors that modulate local urban populations of *Ae. aegypti*. In these urban communities, we have previously observed the importance of water storage practices, waste management, and larvicides modulating entomological indices (12, 15). Additionally, urban mosquito productivity has been associated with factors such as container type, environmental conditions, climate, and seasonality (5, 16, 17). However, limited information is currently available for seasonal mosquito abundance patterns in rural communities in Central America. Other regions have observed that density of children, lot size, water containers, and water storage practices impact mosquito abundance (18–21).

Most studies in Nicaragua and elsewhere have focused on *Ae. aegypti* populations found in urban environments, largely because this mosquito is commonly associated with urban settings. It is thought that urbanicity issues such as a dilapidated infrastructure, erratic supply of water or water intermittency, poor sanitation services, and increasing human population density can produce ideal conditions for high *Aedes* mosquito densities. However, the importance of *Ae. aegypti* and the diseases it transmits has also been demonstrated in rural areas (18–21). Nonetheless, the definition of what is “urban” or “rural”, and where urbanicity starts and finishes can vary in both time and place, making comparison difficult. For this reason, it is critical to compare areas where entomological measurements are performed concurrently within a well-defined spatiotemporal framework.

This project was carried out as part of the Asian-American Centers for Arbovirus Research and Enhanced Surveillance (A2CARES) of the NIH Centers for Research on Emerging Infectious Diseases (CREID) network. The objective of this study was to determine how *Ae. aegypti* entomological indices differ in urban and rural areas and identify which breeding sites were associated with higher productivity by area. We found that all entomological indices of *Ae. aegypti* were higher in “rural” as compared to “urban” areas in both dry and rainy seasons across multiple years, challenging the concept of *Ae. aegypti* as an urban mosquito.

## Material and Methods

### Study area

**Figure 1:**
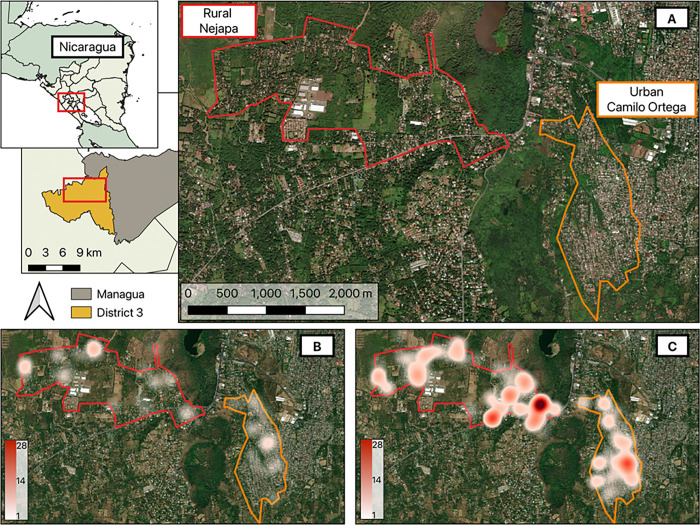
Entomological collections conducted in dry and rainy seasons of 2022–2023 in communities of District 3 of Managua, Nicaragua. **A**) Rural (Nejapa) and Urban (Camilo Ortega) communities of District III. **B**) Adult *Aedes aegypti* collections of the combined dry seasons of 2022–2023. **C**) Adult *Ae. aegypti* collections of the combined rainy seasons of 2022–2023. Maximum adult *Ae. aegypti* collection threshold was set at 28 for both dry and rainy seasons. Map was generated using Quantum GIS (QGIS 3.30) using freely available administrative boundaries and OpenStreetMap (59) for satellite imagery.

This study was conducted in District III of Managua, Nicaragua (Fig. 1), during the dry season (February-March) and the rainy season (October-November) in both 2022 and 2023. The capital city of Managua comprises seven districts, of which District III is the largest, with an area of 73.2 km^2^ and a population of ~ 187,000 inhabitants. It is located 100–400 meters above sea level, with a predominantly tropical climate characterized by two well-defined seasons (dry: December-May; rainy: June-November) and temperatures that range between 18 and 40°C (22). We worked in 21 neighborhoods of District III, within the catchment area of two health posts in Camilo Ortega and Nejapa areas. Camilo Ortega is an urban area with ~ 26,000 inhabitants, and Nejapa is designated as a rural area, with ~ 11,000 inhabitants. We used the official classification of the Nicaraguan Ministry of Health (MOH) to classify urbanicity within our sites (23).

### Study design and samples size

The entomological surveillance program established is part of an ongoing community-based cohort study evaluating arboviruses. The parent cohort has > 2,000 participants living in > 1,000 households who undergo a yearly evaluation of arboviral infections and disease across a gradient of urbanicity. A total sample size of 468 households (234 per urban and rural communities) was estimated based on a 20% infestation rate during the dry season, at 95% confidence and a margin of error of 5%. We assumed a 5% non-response rate due to community members not available for a total roundup of 500 households (250 per community). These 500 households were randomly selected from the parent cohort of 1,000 households for serological surveillance. Household selection was performed using a framework for patches of risk based on landscape features (*i.e.*, vegetation, house rooftops, and open areas). To create a spatially representative sampling design, we used satellite imagery to establish the rural and urban study areas in District III, using impervious surfaces (man-made structures) as a proxy for inhabited sampling locations, which were then selected randomly, taking into account close-pair points and allowances for misclassification and inaccessibility. Households from the previous visits that were found closed or un-inhabited were replaced with the closest house from the parent cohort. A total of 384 (77%) households had the complete set of 4 measurements. Of the 116 (23%) households that were replaced, the most common reason for drop-out was participant migration (> 60%).

### Entomological surveillance

Field visits were conducted from 8 am to 5 pm Monday to Friday, with occasional Saturday visits for households that were not able to be visited during the weekdays. The MOH carried out routine *Ae. aegypti* control and surveillance activities at the study sites. Every two months, MOH vector control field technicians made inspection visits to houses to eliminate immature forms of vectors by placing larvicide in clean water containers in the household. Complementary activities carried out included pesticide fumigation and community clean-up campaigns. We scheduled our activities to avoid being in a community when MOH vector control efforts were being performed. All study data collected underwent a double entry procedure for quality assurance.

#### Immature mosquito collections:

Collection of larvae and pupae was conducted by inspecting indoor and outdoor containers that could hold water. This inspection was initiated outdoors and finished indoors; in each site, field technicians advanced from right to left, covering all grounds. For each container, we documented the following characteristics: type, location (inside or outside), use of water (*i.e*., consumption, human use, cleaning, irrigation, nothing), frequency of scrubbing, condition of covering (full, half, or none), material, presence of moss on the walls, and treatment with larvicide. Type of containers was classified into six categories: 1) barrels (volume of ≥ 200 liters of water), 2) cement laundry sinks, 3) tires, 4) buckets (volume of ≤ 20 liters), 5) other useful containers (jugs, animal water dishes, among others), and 6) non-useful containers (pieces of plastic, scrap metal, etc.). Immature mosquito stages, either larvae or pupae, found in containers were collected using a net, Pasteur pipette, and/or basin. The collection procedure lasted 15–20 minutes, depending on the property size and number of containers. The immature stages were stored in jars with 70% alcohol, labeled with the house unique Identifier, and transported to the study entomology laboratory for taxonomic classification. We performed taxonomic classification of larvae and pupae only for *Ae. aegypti* and *Ae. albopictus*.

#### Adult collection:

Adult mosquitoes were collected using backpack aspirators (Prokopacks^®^), with sampling also starting outdoors and moving indoors. The outdoor aspiration procedure was carried out on the exterior walls of the house focusing on plants, water barrels, tires, and animal water dishes. Indoor aspirations were thorough, with all living quarters inspected (bedrooms, living room, kitchen and bathrooms), sampling under, behind and over furniture, beds and closets. Different collection cups were used for indoor and outdoor collections, labeled with the house unique Identifier, and stored in a thermal bag carried by the field worker, maintaining a temperature of 4–8°C to avoid damage to the mosquitoes. All mosquitoes were transported to the laboratory field station and stored at −20°C until further processing. Mosquitoes were separated by species (*Ae. aegypti, Ae. albopictus, Culex quinquefasciatus, Culex coronator, Anopheles albimanus*, others) (24–26), sex (male and female), and engorged state (bloodfed and unfed). This adult aspiration procedure lasted between 10–15 minutes per collection site.

### Entomological indices

For each entomological visit, we calculated the *Stegomyia* indices (House Index, Container Index, and Breteau Index), pupal indices (pupae per person, pupae per container, and pupae per house), and adult indices (adult index, adults per person, adults per house, and females per person). Container productivity was also estimated as the percentage of total *Ae. aegypti* pupae collected by type of container (27–29).

Stegomyia indices are defined as follows:

House Index (HI): (# houses with immature *Ae. aegypti*/total houses inspected) *100

Container Index (CI): (# containers with immature *Ae. aegypti*/total containers per house) *100

Breteau Index (BI): (# of containers with immature *Ae. aegypti*/total houses inspected) *100

Pupal indices are defined as follows:

Pupae per house index (PHI): # of *Ae. aegypti* pupae/inspected houses

Pupae per container index (PCI): # of *Ae. aegypti* pupae/inspected containers

Pupae per person index (PPI): # of *Ae. aegypti* pupae/total population of inspected houses

Adult indices are defined as follows:

Adult index (AI): (# of houses positive for adult *Ae. aegypti*/total of houses inspected) *100

Adults per person (AP): # of adult *Ae. aegypti*/total population of inspected houses

Adults per houses (AH): # of adult *Ae. aegypti*/total houses inspected

Females per person (FP): # of adult *Ae. aegypti*/total population of inspected houses

### Data analysis

Initial descriptive analyses were performed for data exploration. Container productivity analysis was only based on containers positive for *Ae. aegypti* pupae, as it has been shown that this entomological indicator is the most accurate to translate to adult abundance (28, 30). Entomological indices were compared between rural and urban areas using either a *z*-test for two population proportions, two-sample independent *t*-test, or Rate Ratio. Significance was determined with a p-value of < 0.05, and 95% confidence intervals (95% CI) were calculated using OpenEpi (31). We evaluated female adult abundance using a generalized linear mixed model (GLMM) and generalized additive mixed model (GAMM) for count data. We employed mixed models for the potential lack of spatial independence (random effect for households) in our data (32, 33). First, we evaluated mosquito counts assuming a Poisson distribution (variance = mean), then compared the fits with those of overdispersed counts (variance > mean) (34, 35). The Quasi-Poisson and negative binomial (NB) distributions were used to evaluate if variance increased linearly or quadratically with the mean (referred to as NB type 1 and type 2 in the R packages used) (36). All models were generated with R 4.4.1 (R Core Team, Vienna, Austria) using the ‘glmmTMB’ (37) and ‘gamm4’ packages (38).

All models tested main effects for community (2 levels = Urban and Rural), Season (2 levels = Dry and Rainy), and Year (2 levels = 2022 and 2023). Additionally, the GAMM models evaluated if stage 4 larvae (L4), pupae, a mixture of both (L4 + pupae), and containers with water had a non-linear relationship with female *Ae. aegypti* abundance. For a detailed step-by-step procedure, see Additional file: Text S1, which includes further explanation regarding model selection. Data heteroscedasticity was evaluated by plotting the residuals as a function of predicted values for the distribution models. Models were selected based on the lowest Akaike information criterion (AIC), a metric for model selection that balances goodness of fit and the number of parameters (39).

## Results

### Community demographic structure

In each of the four surveys, we inspected 500 houses (250 urban houses and 250 rural houses), for a total of 2,000 inspections. In the urban communities, we observed an average of 5.1 (SD = 2.07) to 5.3 (SD = 2.46) people per house, of which approximately 39–41% were children ≤ 17 years of age. The proportion of adults aged 18–59 remained around 51–52%, and the proportion of adults aged ≥ 60 years ranged from 7 to 9%. In contrast, rural communities averaged 4.4 (SD = 2.01) to 4.7 (SD = 2.36) people per household. Approximately 37–38% of the rural population were children aged ≤ 17 years. The proportion of adults aged 18–59 years was slightly higher than in urban communities, ranging from 55–56%, while adults aged ≥ 60 represented 6–7% of the rural population. No statistical difference between communities by age structure was observed. Within the urban communities, 88–90% of the respondents were female, of which 6–8% did not have formal school studies. In rural communities, 82–84% of the respondents were female, and between 5–6% had no formal school studies (Additional file 1: Table S1).

### Productive pupal containers vary by rural vs. urban area

We inspected a total of 6,727 containers throughout the course of the study, many of which underwent double, triple or quadruple inspections (Table 1). We did not mark containers; as such, we are unable to provide a measurement for container replacement in the communities. In urban communities, during the dry seasons of 2022 and 2023, on average we detected 2.4 (SD = 2.52) to 2.6 (SD = 2.15) containers per household, of which 2.8% (SD = 0.27) to 6.1% (SD = 0.45) were positive for pupae. In rural communities, the mean number of containers inspected was 3.8 (SD = 4.53) in 2022 and 4.2 (SD = 4.61) in 2023, of which between 2.7% (SD = 0.33) and 6.5% (SD = 0.57) were positive for pupae, respectively. During the rainy season of 2022 and 2023, in urban communities, an average of 2.4 (SD = 1.31) to 3.1 (SD = 2.50) containers were found per household, of which between 7.4% (SD = 0.45) and 10.8% (SD = 0.77) were positive for pupae. In rural communities, the average number of containers inspected per household was 3.1 (SD = 2.07) in 2022 and 5.2 (SD = 5.1) in 2023, with 13.8% (SD = 0.81) to 15.5% (SD = 1.49) containing pupae.

In the dry season of 2022, we observed that only 1.6% (8/500) of all households in the study contributed 67.2% (3,268/4,862) of the total larvae and pupae collected. This pattern continued in the 2022 rainy season; 6.2% of households accumulated 63.8% (7,945/12,455) of the documented larvae and pupae. In the dry season of 2023, the distribution was similar: 3.2% of households concentrated 52.8% (3,624/6,861) of the total larvae and pupae collected. In the rainy season of 2023, 11.2% of households accumulated 68.6% of the larvae and pupae collected (11,710/17,076). These households had a high density of immature stages, with ≥ 100 larvae and at least one pupa in each household.

**Figure 2:**
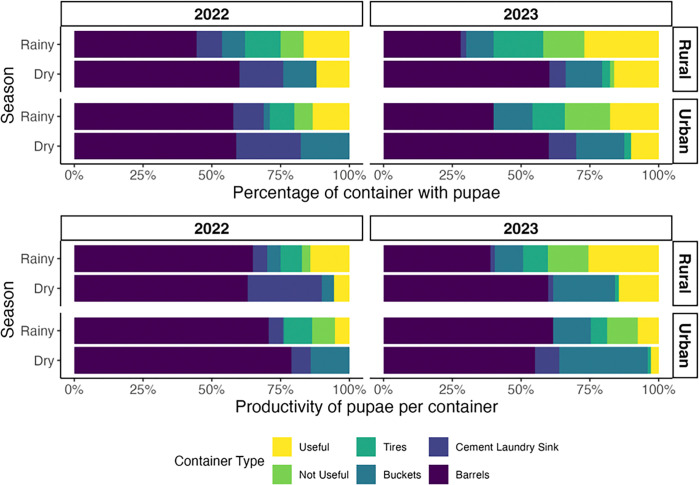
*Ae. aegypti* pupae collected in rural and urban communities of Managua, Nicaragua, during the rainy and dry seasons of 2022 and 2023. **A**) Percentage of containers positive for pupae. **B**) Productivity of pupae per container.

In urban communities, the containers most productive for pupae were barrels (accounting for 55–79% of total pupae) and buckets (0.4–32%) across all seasons and years (Fig. 2A), with cement laundry sinks accounting for 0%–9% of productivity during the dry seasons (Fig. 2B). Similarly, barrels were also the most productive containers in rural communities, with 39–65% of productivity across all seasons and years. However, other types of containers, such as useful and non-useful containers, were documented to contribute 40% of overall productivity during the rainy seasons.

It is important to highlight the importance of water intermittency in the region. Our results show that 51.1% of rural households faced interruptions in water supply, with an average of 6.87 hours (SD = 7.77) per day without running water, while in urban communities, this problem affected 34.8% of households, with average daily interruptions of 3.67 hours (SD = 5.77). This factor has been previously shown to be a risk factor for immature *Aedes* presence in households (15).

### Higher Ae. aegypti abundance in rural communities Immature stages

Throughout the course of the study, a total of 41,254 immature (larvae: 37,255 and pupae: 3,999) *Ae. aegypti* were collected (Table 1). Larval collections in urban communities accounted for 30% of total larvae (n = 11,209), with distinct seasonal patterns of higher abundance during the rainy season 8,269 larvae compared to the dry season 2,940 larvae. Unsurprisingly, a similar pattern was also observed for pupal collections. Pupal collection in urban communities accounted for 26% of total pupae (n = 1,037), with 731 in the rainy season and 306 during the dry season.

In rural communities, we collected 70% (n = 26,046) of total larvae, with 18,305 in the rainy season and 7,741 in the dry season. As in the urban area, pupal collections also had a marked seasonal pattern. Pupal collections in rural communities accounted for 74% of total pupae (n = 2,962) pupae, with 2,226 in the rainy season and 736 in the dry season.

**Figure 3:**
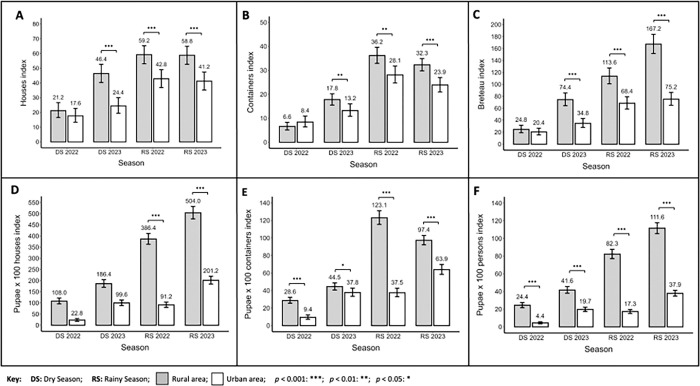
Entomological indices measured in rural and urban communities of District 3 of Managua, Nicaragua. **A**) House Index (HI). **B**) Container Index (CI). **C**) Breteau Index (BI). **D**) Pupae per 100 house index (PHI). **E**) Pupae per container index (PCI). **F**) Pupae per person index (PPI).

We observed that the traditional *Stegomyia* indices of HI (Fig. 3A), CI (Fig. 3B), and BI (Fig. 3C) were statistically higher across all measurements for rural communities compared to urban areas, with the exception of the dry season of 2022 (see Additional file 1: Table S2-S7). Values for rural communities were highest during the rainy season, ranging from 59.2 to 58.8 for HI, 36.2 to 32.3 for CI, and 113.6 to 167.2 for BI in 2022 and 2023, respectively. In contrast, in urban communities for the same seasons, the indices ranged from 42.8 to 41.2 for HI, 28.1 to 23.9 for CI, and 68.4 to 75.2 for BI in 2022 and 2023, respectively. Additionally, the pupal indices of PHI (Fig. 3D), PCI (Fig. 3E), and PPI (Fig. 3F), were also statistically higher for rural communities across all measurements. Values for rural communities were highest during the rainy season, ranging from 386.4 to 504 for PHI (no statistical difference for both summer seasons), 123.1 to 97.4 for PCI, and 82.3 to 111.6 for PPI in 2022 and 2023, respectively. In urban communities, these values were 91.2 to 201.2 for PHI, 37.5 to 63.9 for PCI, and 17.3 to 37.9 for PPI in 2022 and 2023, respectively.

#### Adult Ae. aegypti collections

We collected a total of 1,468 adult *Ae. aegypti* (Table 2). In urban communities, we collected 38% of all adult females (n = 274), of which 23% (n = 63) were collected in the dry season and 77% (n = 211) in the rainy season. In rural communities, we collected 62% of all adult females (n = 441) females, of which 27% (n = 120) were collected in the dry season and 73% (n = 321) in the rainy season. In urban communities, we collected 41% (n = 309) of all adult males, of which 26% (n = 81) were collected in the dry season and 74% (n = 228) in the rainy season. Rural communities accounted for 59% (n = 444) of the total adult males, of which 28% (n = 126) were during the dry season and 72% (n = 318) during the rainy season.

We observed distinct patterns of mosquito infestation and abundance in both urban and rural communities. The best fit models for the GLMM showed a Negative Binomial 2 type distribution with an AIC of 4341.6. We observed a statistically significant difference in the overall abundance of female mosquitoes, with urban communities having 36.5% less females than rural communities (OR = 0.635, 95%CI 0.53–0.77, p < 0.001). This statistically significant difference was observed across seasons and years (Additional file 1: Text S1-GLMM best fit model), with a consistently higher statistically significant abundance of female mosquitoes in rural communities.

**Figure 4:**
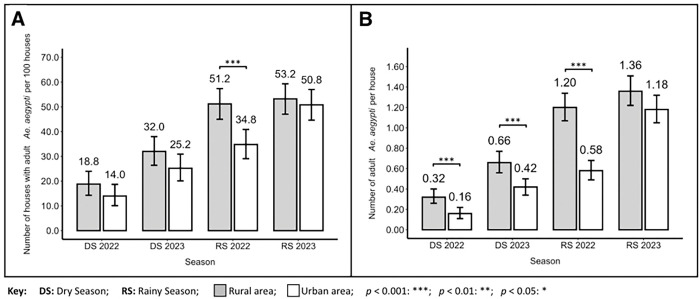
Adult *Aedes aegypti* household indices for rural and urban communities of District III of Managua, Nicaragua. **A**) Households with adult *Ae. aegypti* (AI). **B**) Adult *Ae. aegypti*per house (AH).

Regarding the proportion of houses positive for adult *Ae. aegypti* (AI) (Fig. 4A), we observed that during the dry seasons, no significant differences were detected between rural and urban communities in 2022 (Rural: 18.8% and Urban: 14.0%) and 2023 (Rural: 32.0% and Urban: 25.2%). However, in the rainy season of 2022, we noted a higher proportion of rural houses positive for adult *Ae. aegypti* (Rural: 51.2% and Urban: 34.8%, *z* = 3.704, p = 0.0002). Nonetheless, in the rainy season of 2023, the proportion of houses positive for adult *Ae. aegypti* was similar between rural and urban areas (Rural: 53.2% and Urban: 50.8%). With respect to the number of adult *Ae. aegypti* collected per house (AH) (Fig. 4B), we observed a significantly higher number of adults in rural compared to urban houses in the dry seasons of 2022 (Rural: 0.32 (SD = 0.818) and Urban: 0.16 (SD = 0.427), *t* = 2.74, df = 498, p = 0.006) and 2023 (Rural: 0.66 (SD = 1.338) and Urban: 0.42 (SD = 0.987), *t* = 2.28, df = 498, p = 0.02). We observed a similar pattern in the rainy season of 2022 with a significantly higher number of adults in rural houses (Rural: 1.2 (SD = 2.692) and Urban: 0.58 (SD = 1.07), *t* = 3.38, p < 0.001). However, in the rainy season of 2023, comparable results were observed in rural and urban houses (Rural: 1.36 (SD = 2.685) and Urban: 1.18 (SD = 2.079)).

**Figure 5:**
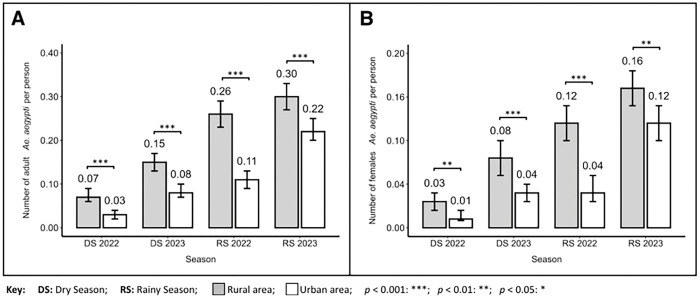
Adult *Aedes aegypti* per person indices for rural and urban communities of District III of Managua, Nicaragua. **A**) Adult *Ae. aegypti* per person (AP). **B**) Adult female *Ae. aegypti*per person (AF).

In relation to the number of adult *Ae. aegypti* per person (AP) (Fig. 5A), we observed 2.4 (95%CI = 1.62–3.47, p < 0.0001) times higher vector density in rural communities compared to urban areas during the dry season of 2022. In the dry season of 2023, rural communities had 1.8 (95%CI = 1.40–2.29, p < 0.0001) times higher vector density than urban communities. This same pattern of higher vector abundance in rural communities was also observed in 2022 (2.3; 95%CI = 1.91–2.84, p < 0.0001) and 2023 (1.4; 95%CI = 1.16–1.58, p = 0.0001).

Finally, a higher density of female *Ae. aegypti* per person (FP) (Fig. 5B), was detected in rural communities compared to urban communities during the dry seasons of 2022 (2.1, 95%CI = 1.20–3.86, p = 0.009) and 2023 (2.2; 95%CI = 1.53–3.15, p < 0.0001). The same was observed in the rainy seasons, with rural communities having a higher female vector density in both 2022 (2.8; 95%CI = 2.04–3.08, p < 0.0001) and 2023 (1.4; 95%CI = 1.11–1.70, p = 0.004). Additionally, as a whole, we observed a trend towards an increase of 1.72 (95% CI = 1.46–2.02, p < 0.0001) times the abundance of female *Ae. aegypti* in 2023 compared to 2022.

### Pupal thresholds for female Ae. aegypti

**Figure 6:**
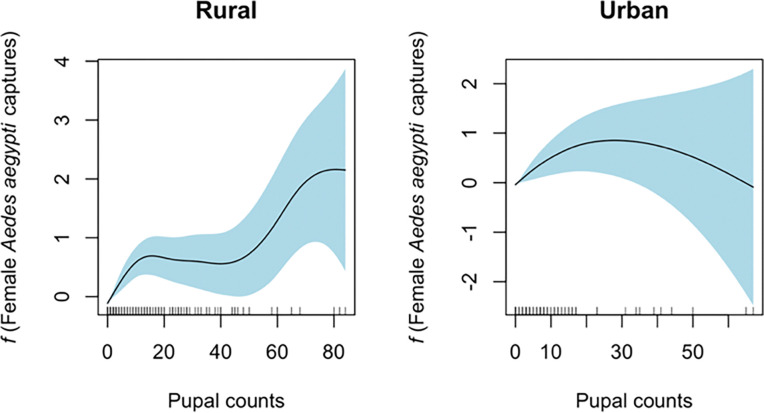
Generalized Additive Mixed Model (GAMM) of pupal counts and female *Ae. aegypti* captured in rural and urban communities of District 3 of Managua, Nicaragua.

We also evaluated the non-linear relationship (smooth terms) of L4 and/or pupae and water-holding containers with the abundance of female *Ae. aegypti*. The models showed that the immature variable of pupae had the best fit (AIC = 4599.8) for female mosquito abundance, with statistical significance of the pupae smooth term (edf = 3.48, Chi^2^ = 46.07, p < 0.001, R^2^ adjusted = 0.126). For the effect of containers with water, we did not observe statistical significance of the smooth function (p = 0.09); however, we observed a linear increase, which would imply a consistent positive association between total containers with water and female adults in the household. Next, we modeled pupae by community. In the rural communities, we observed that the relationship between female adult *Ae. aegypti* and total pupae increased in waves in response to the total number of females in the household. Specifically, there was an initial increase in pupae abundance from 1 to around 18 pupae, followed by a plateau between approximately 19 to 50 pupae, finalizing in a sharp increase beyond 50 pupae. This pattern suggests that as the count of pupae increases, there is an initial positive effect on number of female *Ae. aegypti*, followed by a stable phase, and then a strong increase, indicating possible thresholds in the effect of pupal abundance on the number of females in the household (Fig. 6A). Our model prediction for adult female counts shows that from 1 to 13 pupae, we have a predicted increase from 0.24 (95%CI = 0.20–0.29) to 0.49 (95%CI = 0.36–0.66) females, a plateau of 0.49 to 0.48 (95%CI = 0.29–0.79) females from 13 to 41 pupae, and an increase to 2.03 (95%CI = 0.35–11.83) females from 42 to 84 pupae. In urban communities, we observed a moderate increase of the smooth peaking at 20 pupae, followed by a consistent decrease, suggesting that increases in pupal counts may not correspond to higher female captures in urban settings (Fig. 6B).

## Discussion

*Ae. aegypti* continues to be a major public health threat in tropical regions and an emerging threat to other parts of the globe due to its ecological range expansion as a consequence of climate change (1, 4, 40). With increasing dengue epidemics globally and billions of people at risk of arbovirus infection, it is critical to gain in-depth knowledge of the local distribution of *Ae. aegypti* and the risk that communities face. In particular, a deeper understanding is needed of communities that are not usually viewed as key for *Ae. aegypti*, such as rural areas (41, 42). Our results in District III of Managua, Nicaragua, clearly show that *Ae. aegypti* had higher abundance in all traditional *Stegomyia* indices and higher mosquito densities in rural as compared to urban communities. Interestingly, households that were positive for adult presence had similar results in both rural and urban communities. These results emphasize the importance of evaluating *Ae. aegypti* infestation and disease transmission in areas that have traditionally not be considered ideal sites for *Ae. aegypti*, as this information can inform public health decision-makers.

Our results highlight significant seasonal and spatial variations in container availability and pupal presence across urban and rural communities. Urban areas had fewer water-holding containers per household, yet pupal positivity was comparable or slightly lower than in rural settings, which suggests that both areas are ideal for mosquito development. Rural households, with greater reliance on water storage, exhibited higher pupal positivity, particularly during the rainy season. A similar result was observed in a longitudinal study in Kenya, with higher pupal density per container in rural sites (43). However, this contrasts with results observed in Colombia, where urban communities showed significantly higher pupal densities in containers than rural communities (21), and Sri Lanka, where higher container indices were reported in urban and suburban areas compared to rural areas (44). The results underscore the ecological differences found by geographic region and the plasticity of *Ae. aegypty* to adapt to changing environments. These highlights the importance of considering local environmental, socio-economic, and behavioral factors at a fine scale when designing vector control strategies.

During the 2022 and 2023 dry seasons, barrels, cement laundry sinks, and buckets were the most productive pupal habitats across all households. In urban areas, these containers accounted for nearly all pupae collected, while in rural areas, they contributed to the majority. Similar trends have been reported in Mexico and Argentina, where large water storage tanks dominated pupal production (45, 46). In the rainy seasons of both years, these container types remained the primary sources of pupae in urban areas, with tires, discarded containers, and useful containers also playing a role. In rural areas, barrels, cement laundry sinks, and buckets were still dominant, though alternative containers, including discarded items, gained importance. Observations from Sri Lanka align with our findings, emphasizing the significance of non-degradable garbage and tires as mosquito breeding sites (47). These results suggest the need for tailored intervention strategies to be implemented by season; while the dry season should focus on traditional water storage containers, the rainy season demands greater attention to eliminating discarded items in outdoor spaces. Additionally, overall we observe very low positive rates of water-holding containers with pupae regardless of rurality, which suggests that targeted elimination of key-breeding sites might be a viable control strategy for District III of Managua.

Our observations over two consecutive years and across both rainy and dry seasons indicate that traditional *Stegomyia* indices in rural communities were significantly higher than those in urban areas. This suggests that rural environments not only support the reproduction of *Ae. aegypti* but also offer more available habitats and favorable conditions for their productivity, resulting in higher container indices throughout the year. Similar findings have been reported in other regions. For instance, a study in Zanzibar, Tanzania, found that rural settings had higher numbers of infested containers, as well as greater counts of *Ae. aegypti* immature forms and pupae compared to urban areas (48). Additionally, higher House and Breteau indices were recorded in rural settings. In contrast, a study in Cambodia did not observe differences in *Stegomyia* indices between rural and urban communities (49). Furthermore, several studies have shown a higher abundance of *Ae. aegypti* in urban areas (21, 50), as it is thought that urbanization might favor the proliferation of this vector (51). These differences across studies might be related to several factors, such as variation in enviromental conditions, human behaviour, and mosquito control practices. Our pupal indices showed that over both rainy and dry seasons, *Ae. aegypti* populations are sustained, and even though the indices are lower during the dry season, they still are within the 50–150 PPI threshold and pose a threat for disease transmission (52). Another interesting observation is that a few houses in our study site contributed the vast majority of total pupae collections. This pattern was not observed in adult collections, which were more uniform across areas with high densities of adult *Ae. aegypti* in both years and seasons, potentially because of the dispersal of mosquitoes due to flight range (53). This could suggest that tackling key hot spots for breeding site (source) reduction might have a larger impact on vector control than area-wide management. It is undeniable that rural areas play a role in sustaining *Ae. aegypti* populations and are at risk of disease transmission; as such, *Ae. aegypti* vector control should also focus on rural communities.

We observed a complex non-linear relationship between *Ae. aegypti* pupal abundance and female mosquito counts in both urban and rural communities. In rural communities, the GAMM models showed that pupae were a significant predictor of female *Ae. aegypti* populations, suggesting specific thresholds where accumulation of pupae leads to an increase in adult female mosquitoes, as observed in the initial rise followed by a plateau and then a sharp rise again above 50 pupae, pointing to a threshold effect. However, in urban communities, this effect was only observed in the initial rise to 20 pupae, before a decline in female abundance. This threshold effect could be modulated by density-dependent effects of pupae, such as pupal density and *per capita* growth, as previously observed in a laboratory setting as a non-linear relationship (54). Our results suggest that after reaching a pupal density of 20 pupae per container in urban areas, the impact on abundance of female adults will be limited (55–57). However, in rural communities, containers that may allow > 50 pupae per container would have enough resources to sustain the full development of more female mosquitoes. This also showcases the need for water-management in rural communities to avoid this threshold of pupal production. We also observed that the number of water-holding containers had a positive linear relationship trend with the total count of female adults, as we had observed previously in Managua (14). Our results highlight the potential ecological and environmental differences between rural and urban settings that influence mosquito abundance dynamics.

Our study reveals some of the intricacies of urban and rural communities in two distinct seasons across two years of data collection. One of the limitations of our study was that the collection only occurred at specific time points; thus, the lack of longer-term temporal data prevents a deeper analysis of the ecological dynamics of these urban and rural communities. However, we compensate for this limitation with a robust sample size and a thorough entomological sampling of the households surveyed. We believe that even though our temporal collection events are restricted to seasons, there is enough evidence to highlight the importance of rural communities in sustaining *Ae. aegypti* populations and the potential to drive arboviral disease transmission in such areas. However, during this time-period, our mosquito-based arbovirus surveillance only yielded positive pools in the urban communities (58). We also acknowledge that this study did not evaluate non-household breeding sites, which can also play a role in sustaining mosquito populations in the area. We are currently evaluating the impact of non-household sites as drivers of population dynamics in District III. Of note, in the 2023 rainy season, we recorded for the first time *Ae. albopictus* within the communities of District III, showcasing the need for further surveillance efforts to evaluate the impact that *Ae. albopictus* might have in the ecology of *Ae. aegypti* and the transmission of arboviruses.

## Conclusion

Our results demonstrate that rural communities had a higher density of adult *Ae. aegypti* and traditional *Stegomyia* indices in comparison to urban ones. We also observed that barrels, cement laundry sinks, and buckets should be treated as key container habitats within a vector control strategy, especially in communities facing water supply interruptions, where water storage is indispensable in dry and rainy seasons. Non-useful containers, tires, and animal water bowls were also found to be key productive containers during the rainy season. Additionally, we observed distinct threshold patterns for pupal density as predictors of adult female abundance in rural vs urban settings; further elucidation of these ecological patterns might be used for vector control strategies. We hope that this study can help guide public health control activities within the region, underscoring the importance of rural areas for *Ae. aegypti* propagation.

## Figures and Tables

**Table 1:** Entomological collections of immature *Ae. aegypti* in urban and rural settings of Managua, Nicaragua, during dry and rainy seasons of 2022 and 2023.

Study site	Season	Year	# Cont. ^a^ inspected	# Cont. + pupae	Larvae	Pupae
**Rural**	Dry	2022	943 (3.8)	25 (2.7%)	3,303 (12.7%)	270 (9.1%)
	Rainy	2022	785 (3.1)	108 (13.8%)	7,684 (29.5%)	966 (32.6%)
	Dry	2023	1.047 (4.2)	68 (6.5%)	4,438 (17.0%)	466 (15.7%)
	Rainy	2023	1.294 (5.2)	200 (15.5%)	10,621 (40.8%)	1,260 (42.5%)
		*Total*	*4.069*	*401*	*26,046 (100.0%)*	*2,962 (100.0%)*
**Urban**	Dry	2022	604 (2.4)	17 (2.8%)	1,232 (11.0%)	57 (5.5%)
	Rainy	2022	608 (2.4)	45 (7.4%)	3,577 (31.9%)	228 (22.0%)
	Dry	2023	659 (2.6)	40 (6.1%)	1,708 (15.2%)	249 (24.0%)
	Rainy	2023	785 (3.1)	85 (10.8%)	4,692 (41.9%)	503 (48.5%)
		*Total*	*2.656*	*187*	*11,209 (100.0%)*	*1,037 (100.0%)*

a Cont. = Containers; in () average containers per households unless stated differently

**Table 2:** Entomological collections of adult *Ae. aegypti* in urban and rural settings of Managua, Nicaragua, during dry and rainy seasons of 2022 and 2023.

Study site	Season	Year	Females (Adult)	Males (Adult)
**Rural**	Dry	2022	33 (7.5%)	48 (10.8%)
	Rainy	2022	142 (32.2%)	158 (35.6%)
	Dry	2023	87 (19.7%)	78 (17.6%)
	Rainy	2023	179 (40.6%)	160 (36.0%)
		*Total*	*441 (100%)*	*444 (100%)*
**Urban**	Dry	2022	18 (6.6%)	22 (7.1%)
	Rainy	2022	58 (21.2%)	87 (28.2%)
	Dry	2023	45 (16.4%)	59 (19.1%)
	Rainy	2023	153 (55.8%)	141 (45.6%)
		*Total*	*274 (100%)*	*309 (100%)*

## Data Availability

Data set is available in ZENODO: https://doi.org/10.5281/zenodo.14861397and R code can be found at: https://github.com/jgjuarez/UrbanRuralManagua
